# Decoding Breast Cancer: Emerging Molecular Biomarkers and Novel Therapeutic Targets for Precision Medicine

**DOI:** 10.3390/ijms27010138

**Published:** 2025-12-22

**Authors:** Dámaris P. Intriago-Baldeón, Eduarda Sofía Pérez-Coral, Martina Isabella Armas Samaniego, Vanessa I. Romero, Juan Carlos Pozo Palacios, Gabriele Davide Bigoni-Ordóñez

**Affiliations:** 1 Grupo de Investigación en Biomedicina Experimental y Aplicada, Facultad de Ciencias de la Salud, Universidad Internacional SEK (UISEK), Quito 170120, Ecuador; damaris.intriago@uisek.edu.ec (D.P.I.-B.); eduarda.perez@uisek.edu.ec (E.S.P.-C.); 2 Escuela de Medicina, Colegio de Ciencias de la Salud, Universidad San Francisco de Quito, Quito 170901, Ecuador; miarmas2002@gmail.com (M.I.A.S.); vromero@usfq.edu.ec (V.I.R.); 3 Instituto de Microbiología, Colegio de Ciencias Biológicas y Ambientales, Universidad San Francisco de Quito, Quito 170901, Ecuador; 4 Facultad de Ciencias Médicas Eugenio Espejo, Campus Cuenca, Universidad UTE, Cuenca 010109, Ecuador; 5 Carrera de Laboratorio Clínico, Facultad de Ciencias Médicas, Universidad de Cuenca, Cuenca 010107, Ecuador; 6 Grupo de Investigación en Salud Sexual y Reproductiva, Facultad de Ciencias Médicas, Universidad de Cuenca, Cuenca 010107, Ecuador

**Keywords:** breast cancer, molecular subtypes, molecular biomarkers, therapeutic targets, precision medicine

## Abstract

Breast cancer is the most frequent gynecological malignancy and the main cause of cancer death in the female population worldwide. One of the most significant challenges in its clinical management is the molecular heterogeneity of malignant breast tumors, which is reflected in the current molecular classification of these entities. In each of these tumor molecular subtypes, distinct genetic alterations are involved, and several intracellular signaling pathways contribute to defining their biological identity and clinical response. This literature review summarized the main classic and emerging biomarkers in breast cancer, along with the therapies associated with them. There are several classic biomarkers associated with this disease, such as estrogen and progesterone receptors, the HER2 receptor, and the Ki-67 cell proliferation marker. Given the limitations of these biomarkers, new biomarkers have been identified, including the *TP53* tumor suppressor gene, the EGFR, different types of RNAs, plus epigenetic and immunological biomarkers. The integration of classic and emerging biomarkers along with new therapeutic targets in the clinical practice has promoted a thorough understanding of the high molecular complexity of breast cancer and the development of precision medicine strategies which increase the chances of therapeutic success.

## 1. Introduction

Breast cancer is one of the most frequent and challenging malignant neoplasms worldwide. It is defined as a heterogeneous group of diseases characterized by the uncontrolled growth and division of breast cells with molecular alterations [1]. This excessive cell proliferation results from the acquisition and integration of several hallmarks of cancer, such as self-sufficiency in growth signals, inhibition of apoptosis, sustained angiogenesis, and evasion of the anticancer immune response, which together promote the formation of malignant breast tumors with metastatic potential [2,3]. Despite increasing advances in early detection and personalized treatments, the incidence and mortality of breast cancer continue to rise in the female population at the global scale, due to its high molecular complexity and heterogeneity [4,5]. In this context, the identification of molecular biomarkers is highly relevant, since these molecules allow the characterization of molecular subtypes of breast cancer, contribute to the clinical management of the disease, predict tumor response to medical treatments, and help to establish more accurate prognoses [6,7]. Therefore, the search for new biomarkers has been promoted, which, in conjunction with classic biomarkers, provide detailed information on the biological characteristics of malignant breast tumors and favor the development and administration of personalized therapies that are adjusted to the molecular profiles of each patient’s tumors [8,9]. Given the need to gather updated scientific evidence on biomarkers for this malignant neoplasm, this literature review summarized the main classic and emerging biomarkers in breast cancer, along with the therapies associated with them.

## 2. Epidemiology and Risk Factors in Breast Cancer

Breast cancer is the most common malignant neoplasm and one of the leading causes of cancer death among women worldwide. According to recent World Health Organization (WHO) estimates, in 2022, more than 2.3 million new cases were diagnosed, and approximately 670,000 deaths from this cause were recorded [10]. The global burden of the disease continues to rise, with an estimated annual increase of 1–5% in incidence in about half of the countries evaluated [11]. Although mortality rates have declined steadily in countries with a very high Human Development Index (HDI), attributed to improved screening and therapies, critical gaps persist in regions with a low HDI, contributing to higher mortality rates [10,11,12,13]. Moreover, there is a high incidence of breast cancer in Australia and New Zealand, North America, and Northern Europe, while the highest mortality rates are found in Melanesia, Western Africa, and Micronesia/Polynesia [12,13]. Furthermore, it has been projected that, by 2050, there will be more than 3 million new breast cancer cases each year, along with a 68% increase in mortality, if global prevention strategies are not strengthened [11].

There are multiple risk factors associated with the development of breast cancer, which can be classified as modifiable and non-modifiable [14]. Among the non-modifiable risk factors that predispose individuals to developing this disease are being female, which is associated with greater stimulation by hormones such as estrogen and androgens [15], being over the age of 50, as this is related to the accumulation of cellular alterations that may increase the likelihood of carcinogenesis [16,17], having a family history of breast cancer in first-degree relatives [18,19], the presence of mutations in genes such as *BRCA1* and *BRCA2* [20,21], belonging to specific ethnic groups such as being a non-Hispanic Caucasian or African American woman [22,23,24], having an early onset of menstruation and a late onset of menopause [25], having high breast tissue density [26,27], having a personal history of breast cancer [28], having a previous diagnosis of other benign conditions affecting the mammary glands [29], and having previously undergone radiation therapy [30]. In this context, it is worth mentioning that early pregnancy and breastfeeding are considered protective factors against breast cancer; however, pregnancy at age 35 is associated with an increased risk of developing this disease [31,32,33]. On the other hand, the modifiable risk factors that increase the probability of developing breast cancer are the use of hormone replacement therapies [34,35], the administration of drugs such as diethylstilbestrol during pregnancy [36,37], lack of physical activity, as exercise reduces the risk of developing this disease [38,39,40], overweight and obesity in postmenopausal women [41], high consumption of alcoholic beverages and tobacco [42,43], vitamin deficiencies such as vitamin D [44], exposure to artificial light at night [45,46], high consumption of processed foods in the daily diet [47], and exposure to chemicals that could have carcinogenic effects [48,49].

## 3. Genetic Basis for Breast Cancer

Multifactorial diseases like breast cancer result from the interaction of genetic and non-genetic factors. Identifying these components allows for a better understanding of malignant breast tumor biology and clinical heterogeneity. The most relevant factors are described below:

### 3.1. Non-Genetic Factors

Recently documented cases in multiple patient cohorts reveal an increased risk of developing breast cancer due to diverse non-genetic factors. These factors include environmental factors such as alcohol consumption [50] and hormone replacement therapy [51], hormonal factors such as prolonged estrogen exposure in cases of early menarche or late menopause [52], reproductive factors such as number of children and breastfeeding [53], and metabolic factors, such as sedentary lifestyle and postmenopausal obesity [54]. It has recently been shown that these hormonal, reproductive, metabolic, and environmental factors can induce epigenetic changes [55], such as hypermethylation of tumor suppressor gene promoters such as *BRCA1* [56], *CDH1*, *RASSF1A*, and *PTEN*, or histone modifications (EZH2, HDAC1), which alter gene expression and modulate the effect of genetic variants related to breast cancer risk [57,58]. This interaction among environment, epigenome, and genotype could explain the differences in breast cancer incidence and clinical presentation observed across populations [59].

### 3.2. Genetic Factors

The development of next-generation sequencing (NGS) has promoted the identification of multiple genes related to breast cancer predisposition. The genetic factors can be classified into germline and somatic variants [60]. Germline variants are inherited and are associated with a medium to a high risk of cancer; it is estimated that 5 to 10% of all breast cancers are caused by germline variants. Updated clinical guidelines recommend, at a minimum, evaluating multigene panels that contain *BRCA1*, *BRCA2*, *PALB2*, *TP53*, *PTEN*, *CDH1*, *STK11*, *ATM*, and *CHEK2* to assess the risk of germline variants [61]. Somatic variants are not inherited but significantly influence prognosis and treatment response, depending on the molecular subtype, and are responsible for the majority of cancers [62]. In general, these genetic factors can be classified into high-penetrance variants, intermediate-penetrance variants, and low-penetrance polymorphisms:

- High-Penetrance Variants: These genetic variants can occur in the *BRCA1* and *BRCA2* genes, with cumulative lifetime risks of 55–70% and 45–69%, respectively [63]. However, the risk varies depending on the variant type and the population studied. Other high-penetrance genes are *TP53* (Li-Fraumeni syndrome) [64], *PTEN* (Cowden syndrome), and *CDH1* (hereditary lobular carcinoma). These genes have been recognized as responsible for hereditary cancer predisposition syndromes, in addition to their association with breast cancer [65].
- Intermediate Penetrance Variants: These genetic variants have been identified in genes like *PALB2*, *CHEK2*, and *ATM*, and are known to confer a moderate to high risk of developing breast cancer, although a lower risk than that associated with *BRCA1* and *BRCA2* [66]. The estimated cumulative risk, situated between 20 and 50% for *PALB2* [67] and 20–40% for *CHEK2* [68], relies on factors like family history and demographic group. Regarding *ATM*, certain variations have also been linked to a moderate risk [69].
- Low-Penetrance Polymorphisms: These genetic variants can be analyzed using polygenic risk scores (PRS), which quantify cumulative risk based on hundreds of single nucleotide polymorphisms (SNPs) and adjust the individual risk of breast cancer [70,71].

### 3.3. Alterations in Intracellular Signaling Pathways Linked to Breast Cancer

Germline and somatic mutations, along with epigenetic alterations, can disrupt intracellular signaling pathways that control and regulate cell proliferation and differentiation, ultimately shaping the tumor phenotype [72]. These pathways include:

- PI3K/AKT/mTOR signaling pathway: Mutations in the *PIK3CA* gene described in hormone receptor-positive (HR+) luminal breast carcinomas plus loss of PTEN or hypermethylation of its promoter induce AKT phosphorylation and mTORC1/2 activation [73]. This signaling pathway stimulates cell proliferation and resistance to genotoxic stress. For this reason, this pathway is used as a therapeutic target for PI3K and mTOR inhibitors [74].
- MAPK signaling pathway (RAS/RAF/MEK/ERK): Amplification of receptor tyrosine kinases (RTKs), such as epidermal growth factor receptor-2 (HER2/ERBB2) and fibroblast growth factor receptor-1 (FGFR1), mutations in RAS/BRAF, or cross-activation with the PI3K/AKT signaling pathway induces cell proliferation, improved cell migration and suppression of apoptosis (associated with aggressive phenotypes of breast cancer such as triple-negative (TNBC) and HER2-positive) [75]. Simultaneous activation of the MAPK and PI3K/AKT signaling pathways induces adaptive therapeutic resistance [76].
- Wnt/β-catenin signaling pathway: Wnt overactivation due to *SFRP1* hypermethylation or *CTNNB1* mutations leads to nuclear accumulation of β-catenin, which in turn regulates the expression of genes associated with cell plasticity and metastasis. This molecular mechanism has been identified in basal and metaplastic tumors [77]. The interaction of this pathway with the PI3K/AKT and MAPK signaling pathways contributes to tumor heterogeneity and treatment resistance [78].

## 4. Molecular Basis for Breast Cancer

### 4.1. Establishment of Molecular Subtypes of Breast Cancer

From a biological point of view, malignant breast tumors are clinically heterogeneous entities, and their molecular classification has enabled the identification of subtypes with distinct prognostic and therapeutic profiles [79]. Malignant breast tumors have been classified into several intrinsic molecular subtypes based on the analysis of their mRNA expression profiles. In 2000, the study conducted by Perou et al. established four intrinsic molecular subtypes of breast cancer based on gene expression data obtained from complementary DNA (cDNA) microarrays that analyzed 8102 genes from 65 human breast cancer specimens derived from 42 different patients: Luminal, HER2-enriched, Basal-like, and Normal Breast-like [80]. Subsequently, the Luminal subtype was divided into two categories: Luminal A and Luminal B subtypes [81,82]. In addition, it has been established that the Normal Breast-like subtype represents biological samples that were contaminated with healthy breast tissue or healthy germline DNA; therefore, this subtype was excluded from the current molecular classification for the disease [2,14,83].

**Figure 1:**
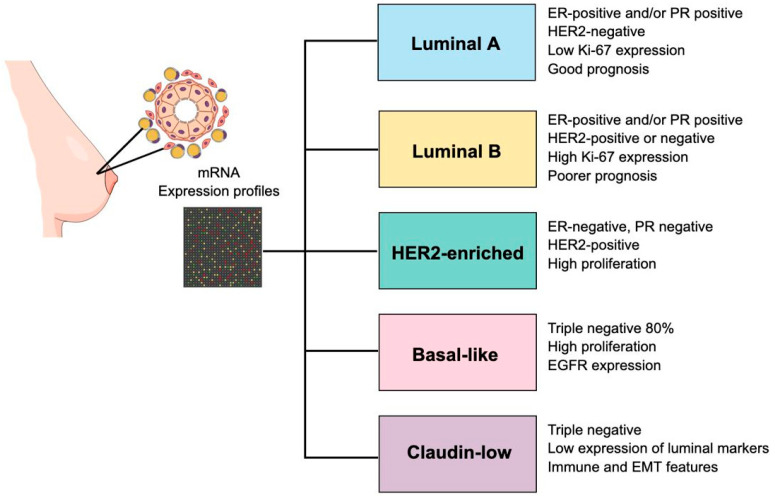
Schematic representation of the five main intrinsic subtypes of breast cancer. Each intrinsic subtype shows distinct molecular signatures and clinical outcomes, ranging from hormone receptor-positive Luminal types to highly aggressive Basal-like and Claudin-low tumors.

Moreover, the results of the Cancer Genome Atlas (TCGA) project, which analyzed tumor and germline DNA samples from 825 patients at the genomic, transcriptomic, and proteomic levels to establish clusters of tumors with molecular similarities, confirmed the existence of four intrinsic subtypes of breast cancer based solely on mRNA expression profiles: Luminal A, Luminal B, HER2-enriched, and Basal-like [84]. Furthermore, a fifth intrinsic molecular subtype called Claudin-low was added to this classification, which was first described in 2007 in a study by Herschkowitz et al. that analyzed the gene expression profiles of 13 breast tumors obtained from murine models of the disease, using cDNA microarrays, and then compared these data with those derived from malignant human breast tumors [85]. Each of these five intrinsic molecular subtypes of breast cancer exhibit unique biological characteristics that directly influence its clinical behavior and prognosis, which are described below (Figure 1).

### 4.2. Characteristics of Breast Cancer Molecular Subtypes

#### 4.2.1. Luminal A

The histological grade of malignant breast tumors belonging to the luminal A subtype is low. These tumors are characterized by the expression of estrogen receptors (ER) and/or progesterone receptors (PR), as well as low-molecular-weight cytokeratins. In addition, these tumors do not express HER2/ERBB2, exhibit low Ki-67 expression, and have a low risk of recurrence. Luminal A malignant breast tumors are the most prevalent, accounting for approximately 50% of all diagnosed breast cancer cases. These tumors are associated with slow clinical progression, a good prognosis, and an excellent response to hormone therapies [86,87,88]. These tumors express a robust luminal genetic signature, including *ESR1*, *GATA3*, *XBP1*, and *FOXA1* genes [84], and show low expression of genes related to cell proliferation [89].

#### 4.2.2. Luminal B

Malignant breast tumors of the luminal B subtype are histologically high grade and tend to have a worse prognosis than luminal A tumors; approximately 20% of all diagnosed cases of breast cancer correspond to this molecular subtype [88,90]. These tumors can be classified into two subgroups: HER2-positive luminal B and HER2-negative luminal B. HER2-positive luminal B tumors express ERs, often overexpress HER2, and may exhibit varying Ki-67 and PR receptor expression. On the other hand, HER2-negative luminal B tumors express ERs and do not express HER2 receptors; in addition, these tumors have at least one of the following characteristics: high expression of the Ki-67 cell proliferation marker, low or no expression of the PR receptor, and a high risk of recurrence [86,87]. Luminal B malignant breast tumors show high expression of genes related to cell proliferation, as well as low-molecular-weight cytokeratins [84,88]. The response of these tumors to hormone therapies and chemotherapy is variable; when these tumors are more biologically aggressive and less sensitive to hormone therapies, combined systemic treatment is usually administered [87,88].

#### 4.2.3. HER2-Enriched

HER2-enriched malignant breast tumors have a high histological grade, are associated with a high proliferative index and an increased risk of metastasis, and have a worse prognosis compared to luminal A and luminal B malignant breast tumors. These tumors show intermediate expression of the luminal genetic signature and do not express the ER and PR hormone receptors. Instead, they are characterized by amplification of the *HER2/ERBB2* gene, located on chromosome 17q12, which leads to overexpression of the HER2 receptor [84,86,88,91]. Approximately, 15% of all diagnosed breast cancer cases correspond to this molecular subtype; these tumors have a high expression of the Ki-67 cell proliferation marker and mutations in the *TP53* gene [88]. It is worth mentioning that therapies targeting the HER2 receptor, such as Trastuzumab and Pertuzumab, which can be administered along with other cancer treatments, such as chemotherapy, have significantly improved disease-free survival (DFS) in patients with this molecular subtype of the disease [92].

#### 4.2.4. Basal-like/Triple-Negative (TNBC)

Malignant basal-like breast tumors represent approximately 15% of all diagnosed cases of breast cancer, have high proliferation rates, and exhibit high expression of basal cytokeratins and epidermal growth factor receptor (EGFR), along with low expression of the luminal A genetic signature. These tumors are characterized by high chromosomal instability and germline mutations in the *BRCA1* gene [84]. Approximately, 80% of these malignant tumors correspond to the TNBC subtype [2]. TNBC is a group of heterogeneous malignant breast tumors that do not express ER and PR hormone receptors and the HER2 receptor. In addition, these malignant tumors exhibit high Ki-67 expression and *TP53* mutations [6,86,88,93]. TNBC tumors are more common in young women, especially the ones of African descent and/or with *BRCA1* gene mutations [6,94,95]. This molecular subtype of breast cancer is associated with an aggressive clinical progression, limited specific treatment options, and a poorer overall prognosis [96].

#### 4.2.5. Claudin Low

Malignant breast tumors that belong to the Claudin-low subtype are generally triple-negative, have a poor prognosis, exhibit low or no expression of luminal differentiation markers, and have a high expression of markers related to the epithelial–mesenchymal transition (EMT), genes associated with the immune response, and cancer stem cell-like features. This molecular subtype of breast cancer has a response to standard preoperative chemotherapy that is intermediate between the one seen in luminal tumors and the one observed in basal-like tumors [97].

## 5. Classic Biomarkers for Breast Cancer

Molecular biomarkers complement traditional clinicopathological characteristics (such as tumor size, histological grade, and lymph node involvement) in guiding personalized therapeutic strategies [98]. They allow the disease to be detected and categorized at early stages, predict therapeutic response, estimate prognosis, and assess the risk of recurrence [99,100]. The so-called classic biomarkers of breast cancer (ER, PR and androgen (AR) hormone receptors, the HER2 receptor, and the Ki-67 cell proliferation marker) were the first ones to be studied and standardized and remain pillars of the clinical management of this disease, despite the emergence of new molecular candidates.

### 5.1. Hormone Receptors

Breast tissue development and differentiation are primarily regulated by estrogen and progesterone, which are hormones that act through the nuclear receptors ER and PR [101]. Both ER and PR are predominantly expressed in luminal epithelial cells, and their immunohistochemical identification is essential for diagnosis, prognosis prediction, and therapeutic selection in breast cancer.

#### 5.1.1. ER

The ER comprises two subtypes: ERα encoded by the *ESR1* gene, located at chromosome 6q25.1-q25.2, and ERβ encoded by the *ESR2* gene, located at chromosome 14q23.2-q23.3. ERα is expressed in 70–75% of malignant luminal breast tumors [101,102,103]. ERβ modulates and counteracts ERα-mediated cell hyperproliferation [6,104]. ER has six functional domains: the N-terminal AF-1-independent A/B domain, the DNA-binding domain (DBD), the hinge region (D), the ligand-binding domain (LBD), the C-terminal domain, and the AF-2 transcriptional activation domain [101]. These domains regulate the transcription of genes, such as *CCND1*, *FOXM1*, *IGF-1*, *MYC*, *BCL2*, and *PGR*, which are involved in cell proliferation and survival [104]. In patients treated with antiestrogens, mutations in the LBD of *ESR1* are associated with therapeutic resistance [105].

#### 5.1.2. PR

The PR exists in two main isoforms, PR-A (94 kDa) and PR-B (114 kDa), both encoded by the PGR gene located at chromosome 11q22.1 but transcribed by alternative promoters [106]. PR-A, which is predominantly nuclear, can inhibit the transcriptional activity of PR-B, which is the primary mediator of progesterone-induced signaling [107]. The alteration in the PR-A: PR-B ratio occurs at the early stages of mammary tumorigenesis, in both tumor and adjacent cells. PR consists of the DBD, the LDB, the amino-terminal and the transcriptional activation domains (AF-1, AF-2, and additional AF-3). PR modulates ERα-mediated estrogen signaling by regulating the expression of genes, such as *RANKL*, *WNT4*, and *CCND1*, which activate signaling pathways, such as PI3K/AKT and MAPK. PR loss is linked to a higher probability of recurrence and development of endocrine resistance, mainly to tamoxifen and exemestane [108].

#### 5.1.3. AR

The AR, encoded on chromosome Xq12, is a ligand-dependent transcription factor that regulates genes involved in proliferation, differentiation, and metabolic balance [109]. Recent evidence has strengthened its role as an emerging biomarker in breast cancer, particularly in TNBC, where AR expression contributes to a distinct molecular phenotype known as the luminal androgen receptor (LAR) subtype [110]. This subgroup shows AR-driven transcriptional activity and unique therapeutic vulnerabilities. Contemporary reviews and translational studies highlight that AR-positive TNBC may exhibit differential prognosis and can respond to androgen-signaling inhibition, supporting ongoing interest in AR-targeted approaches [111]. Together, these findings position AR as an increasingly relevant biomarker with growing clinical and molecular implications in TNBC.

### 5.2. HER2/ERBB2

The HER2/ERBB2 is a tyrosine kinase receptor encoded by the *ERBB2* gene, located at chromosome 17q12, which is composed of three domains: the extracellular domain (ECD) with four subdomains (I–IV), the transmembrane domain (TMD), and the intracellular region [112]. Receptor activation through homo- or heterodimerization induces autophosphorylation of tyrosine residues, which triggers signaling pathways, such as MAPK and PI3K/AKT/mTOR [112,113]. The HER2 receptor is amplified or overexpressed in 15–30% breast carcinomas, especially in those that are more aggressive and have a poor prognosis [113]. Intratumoral heterogeneity in HER2 receptor expression may reduce the efficacy of targeted therapies [114]. Point mutations that affect the structure of the ECD or the intracellular tyrosine kinase region, independent of amplification or overexpression, are associated with therapeutic resistance [115,116,117,118].

### 5.3. Ki-67 Cell Proliferation Marker

Ki-67 is a nuclear antigen of approximately 359 kDa encoded by the *MKI67* gene, which is actively expressed during the G1, S, G2, and M phases of the cell cycle, but is absent in quiescent cells (G0) [7,119]. It is overexpressed in approximately 16.67% of breast carcinomas, particularly in enlarged hyperplastic lobular units [120]. In oncology, it is used as an index of cell proliferation, and it is associated with a poor prognosis; expression above 60% is associated with a higher risk of recurrence and lower survival rates [121]. It is also used as a predictive biomarker of response to endocrine therapies. For example, an increase in its expression after treatment is associated with a poorer response and lower recurrence-free survival, while high baseline expression does not necessarily predict poor response [121]. Despite its clinical utility, there are limitations in the consensus on the cutoff point to distinguish between low- and high-risk tumors, in interobserver variability, and in the standardization of quantification methods, traditionally performed by microscopy [122,123,124].

## 6. Emerging Molecular Biomarkers for Breast Cancer

**Figure 2:**
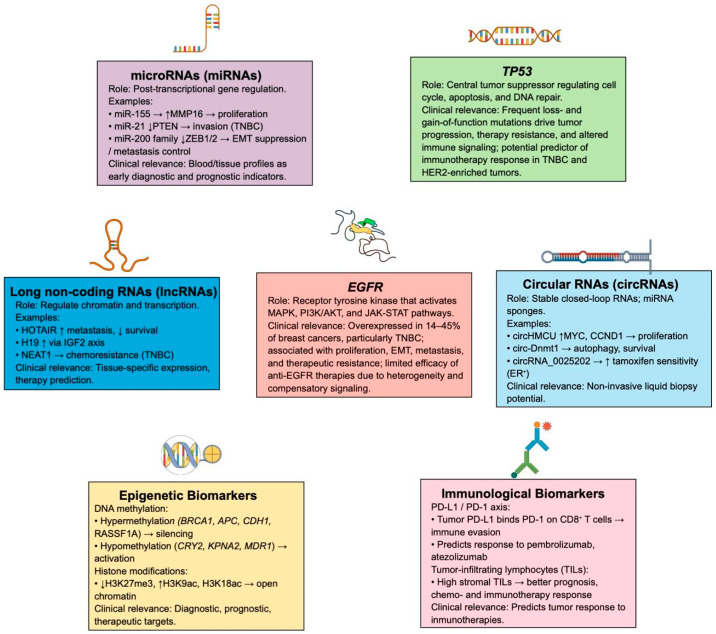
Summary of the main emerging molecular biomarkers in breast cancer. Emerging biomarkers include non-coding RNAs, epigenetic, genomic, and immune markers that collectively contribute to tumor progression, therapeutic response, and precision oncology.

A wide range of molecular alterations have been identified as potential biomarkers for breast cancer, including genetic mutations, aberrant receptor signaling, dysregulated expression of non-coding RNAs, epigenetic modifications, and immune-related markers. These biomarkers reflect tumor heterogeneity and have important implications for diagnosis, prognosis, and therapeutic decision-making (Figure 2).

### 6.1. TP53 Tumor Suppressor Gene

*TP53* is a tumor suppressor gene located on chromosome 17p13.1 that encodes the p53 transcription factor, which is composed of a transactivation domain (TAD), a DBD, a tetramerization domain, a proline-rich domain, and a regulatory domain [125]. *TP53* is the most frequently mutated gene in human cancer, with both loss-of-function and gain-of-function mutations that can alter cell cycle regulation, apoptosis, senescence, DNA repair, and accumulate genetic alterations [126,127]. Therefore, malignant tumors harboring *TP53* mutations are associated with rapid progression, resistance to conventional therapies, and poor prognosis.

Approximately 85% of women carrying *TP53* mutations eventually develop breast cancer, either due to germline (5–8%) or somatic (37%) mutations; these mutations predominate in triple-negative, HER2-enriched, and basal-like breast cancers, and they are usually located in exons 5–8 [126]. In addition, certain *TP53* mutations could induce immunogenicity in breast cancer through the regulation of several p53-mediated signaling pathways, which could be associated with a better prognosis in *TP53*-mutated malignant breast tumors; this result implies that *TP53* mutation status could be considered as a potential biomarker to classify patients who might be responsive to immunotherapies [128].

### 6.2. EGFR/HER1/ERBB1

The EGFR is a tyrosine kinase receptor consisting of an ECD, a TMD, and an intracellular region with tyrosine kinase activity [129]. Its activation occurs after the binding of ligands that induce its dimerization, autophosphorylation, and subsequent activation of the MAPK, PI3K/AKT, and JAK-STAT signaling pathways [76,129]. EGFR signaling can be amplified by the increased expression of ligands, such as TGF-α, Heparin-Binding EGF-Like Growth Factor (HBEGF), and amphiregulin (AREG), which can be produced endogenously in response to lifestyle or environmental factors [130]. It is overexpressed or aberrantly activated in 14–45% of breast carcinomas, especially in TNBC, and it is associated with increased proliferation, resistance to apoptosis, metastasis, EMT, and poor clinical prognosis [131,132,133]. The therapeutic potential of EGFR-targeted monoclonal antibodies and tyrosine kinase inhibitors is limited, with a tendency toward recurrence and the development of intrinsic or acquired resistance [134]. This is mainly attributed to tumor heterogeneity, gain-of-function mutations, chromosomal rearrangements, and aberrant or compensatory activation of signaling pathways [130]. In this context, pharmacological inhibition of EGFR may disrupt its crosstalk with other receptor tyrosine kinases, such as IGF-1R, which promote cell proliferation. Consequently, breast cancer cells may activate compensatory signaling pathways, thereby reducing the efficacy of anti-EGFR therapies [135].

### 6.3. Different Types of RNAs

Non-coding RNAs add an additional regulatory dimension to breast cancer biology. Through their influence on transcription, RNA processing, and protein interaction networks, these molecules help shape tumor behavior and contribute to clinically relevant phenotypes. Among the most extensively studied groups are microRNAs, long non-coding RNAs, and circular RNAs. Each category participates in distinct molecular mechanisms that affect tumor growth, progression, and therapeutic response.

#### 6.3.1. MicroRNAs

MicroRNAs (miRNAs) are short non-coding RNA molecules—typically 15 to 25 nucleotides long—that fine-tune gene expression by pairing with complementary sequences in target mRNAs, promoting their degradation or blocking translation. Through this post-transcriptional regulation, miRNAs participate in biological processes such as epigenetic remodeling, control of protein turnover, and gene silencing. Disruption of miRNA expression or activity can alter key signaling pathways and contribute to cancer initiation and progression [136,137,138]. Functionally, cancer-related miRNAs fall broadly into two subgroups: oncogenic miRNAs (oncomiRs), which become overexpressed and enhance tumor-promoting traits, and tumor-suppressive miRNAs, which restrain cell growth, modulate immune responses, or promote apoptosis, thereby counteracting malignant transformation [139,140].

Clinically, miRNAs have attracted considerable attention because they can be reliably detected in both tissues and body fluids, offering opportunities for minimally invasive diagnostic assays. Numerous studies have reported that women with breast cancer display distinct miRNA expression patterns in blood and tissue samples, which differ markedly from healthy or pre-malignant states [141,142,143,144,145]. These signatures often correlate with tumor subtype: miRNA expression tends to be higher in hormone-receptor-positive tumors and lower in basal-like cancers. Additionally, certain miRNAs show expression patterns associated with *BRCA1*, *BRCA2*, and *TP53*, both before and after chemotherapy [146].

Several individual miRNAs illustrate these roles; for example, miR-155, whose expression is normally restrained by BRCA1/2, promotes proliferation and migration by suppressing SOCS1 and enhancing MMP-16 levels [146,147]. miR-21 is frequently elevated across breast cancer cohorts, particularly in triple-negative cases, where it contributes to tumor aggressiveness in part by downregulating PTEN [146,148]. The miR-200 family (miR-200a/b/c, miR-141, miR-429) regulates epithelial–mesenchymal transitions by inhibiting transcriptional repressors, such as ZEB1/2, although under certain conditions these same molecules can facilitate metastatic spread [146,149,150,151,152,153,154,155].

Furthermore, studies have been conducted to advance miRNA-based therapeutics into the clinical setting, by identifying strategies that could enhance the pharmacological effects of these molecules; for example, a study led by Abdelaal et al. (2023) [156] synthesized and tested a fully modified version of miR-34a (FM-miR-34a) which was conjugated to a synthetically simplistic ligand. This molecule is more stable than its partially modified version, and it exhibited strong silencing of its gene targets; moreover, it inhibited proliferation, migration and invasion of the MB-231 breast cancer human cell line in vitro. In addition, it induced a substantial reduction in tumor growth in mice because of its conjugation to folate (FM-FolamiR-34a); these results suggest that miR-34a could become an anticancer agent with clinical potential.

#### 6.3.2. lncRNAs

Long non-coding RNAs (lncRNAs) are transcripts longer than 200 nucleotides that, despite lacking protein-coding potential, exert regulatory effects at multiple cellular levels [157]. Found in both the nucleus and cytoplasm, lncRNAs modulate gene expression by interacting with DNA, transcription factors, mRNAs, miRNAs, or protein complexes, influencing processes such as chromatin organization, transcriptional activation or repression, and RNA stability [158,159].

Because many lncRNAs exhibit tissue-specific expression patterns and participate in epigenetic regulation of oncogenes and tumor-suppressor genes, they are being explored as candidates for diagnostic and prognostic use. In breast cancer, numerous lncRNAs show characteristic dysregulation. HOX transcript antisense intergenic RNA (HOTAIR) is a well-known example: its overexpression enhances proliferation, promotes EMT, and facilitates metastasis [160,161]. Elevated HOTAIR levels correlate with lymph-node involvement and poorer overall survival (OS), especially in TNBC [162,163,164]. H19, one of the earliest lncRNAs linked to breast cancer, regulates the H19/IGF2 imprinting axis and is frequently upregulated across multiple tumor types [165]. NEAT1 contributes to chemoresistance by sequestering pro-apoptotic miRNAs in TNBC [166]. Likewise, CUPID1 and CUPID2 influence stress-response pathways and hormone-receptor signaling, and their dysregulation has been associated with susceptibility to ER- and PR-positive breast cancers [167].

#### 6.3.3. circRNAs

Circular RNAs (circRNAs) represent a specialized subgroup of non-coding RNAs characterized by a covalently closed loop generated through a back-splicing mechanism that links the 3′ and 5′ ends of exons and/or introns. This circular architecture provides strong resistance to exonuclease degradation, contributing to their stability and making them attractive candidates as biomarkers [168]. Their presence in biological fluids—including plasma and saliva—also supports the development of non-invasive diagnostic approaches [169]. The best-described mechanism of circRNA function is their ability to act as miRNA sponges, sequestering specific miRNAs and thereby modulating entire regulatory networks involved in cancer pathogenesis [170]. However, circRNAs may also interact with RNA-binding proteins, participate in transcriptional regulation, and, in some cases, encode small peptides with biological activity.

A variety of circRNAs have been implicated in breast cancer progression. circHMCU enhances proliferative and metastatic behavior by binding to members of the let-7 miRNA family, which leads to increased expression of *MYC*, *HMGA2*, and *CCND1* [171]. circ-Dnmt1, frequently upregulated in malignant breast cells, promotes autophagy and supports tumor survival; its inhibition reduces cell proliferation and tumor growth in vivo [172]. Conversely, circRNAs, such as circCCDC85A, hsa_circ_0072309, and circVRK1, tend to be downregulated in breast cancer, and their re-expression suppresses proliferation, invasion, and migration [173,174,175]. Additional molecules, including hsa_circ_0006743 and hsa_circ_0002496, are enriched in early-stage breast cancers [176]. Some circRNAs also influence therapeutic outcomes: circRNA_0025202 enhances tamoxifen sensitivity in ER-positive malignant breast tumors [177], while hsa_circ_0000199, which is elevated in TNBC, modulates response to chemotherapy and improves treatment efficacy when suppressed [178].

### 6.4. Epigenetic Biomarkers

In cancer, epigenetic mechanisms such as DNA methylation and histone post-translational modifications are abnormally dysregulated, leading to changes in gene expression without altering the DNA sequence [179,180]. These mechanisms regulate the activation or silencing of oncogenes and tumor suppressor genes and may be involved in the processes of cell proliferation, apoptosis, and differentiation [181]. Recently, there has been an increase in reports of recurrent mutations in genes encoding epigenetic modulators associated with EMT, pluripotency, and drug resistance [182,183]. Therefore, it has been proposed that epigenetic patterns could serve as biomarkers for diagnosis and prediction of prognosis and therapeutic response, as well as potential targets for the development of new pharmacological strategies.

#### 6.4.1. DNA Methylation in Promoter Regions

DNA methylation is a heritable and reversible epigenetic process mediated by DNA methyltransferases, encoded by the *DNMT1*, *DNMT3A*, and *DNMT3B* genes, which catalyze the addition of a methyl group to the carbon-5 of cytosines [184,185]. This event occurs mainly in regions rich in dinucleotides (CpG), such as the promoters and regulators of oncogenes and tumor suppressor genes. On the other hand, demethylation occurs via Ten-Eleven Translocation (TET) dioxygenases, which oxidize 5-methylcytosine (5mC) to generate 5-hydroxymethylcytosine (5hmC) [186,187,188]. In general, cancer cells exhibit global DNA hypomethylation and promoter hypermethylation. Hypomethylation of promoters of oncogenes, such as *CRY2* and *KPNA2*, and drug resistance-associated genes, such as *MDR1*, favors their activation and increases genomic instability, contributing to uncontrolled cell proliferation and metastasis [183]. Hypermethylation in promoters of tumor suppressor genes, such as *BRCA1*, *APC*, *CDH1*, *CCND2*, *CTNNB1*, *FOXA1*, *SOX10*, *p16*, and *RASSF1A*, causes their transcriptional silencing [183,189,190,191]. In breast cancer patients, hypo- and hypermethylation events have been observed during the early stages of the disease. At the same time, changes in methylation patterns have been reported during the transition from healthy mammary tissue to ductal carcinoma in situ (DCIS), with minimal epigenetic modifications between DCIS and the invasive form [179,189].

#### 6.4.2. Histone Modifications

Histone modifications consist of post-translational alterations of the N-terminal tails of histone proteins, including methylation, acetylation, phosphorylation, ubiquitination, and sumoylation. These processes catalyzed by enzymes, such as histone acetyltransferases (HATs), histone deacetylases (HDACs), and histone methyltransferases (HMTs), which modify the structure of chromatin and, therefore, the accessibility of DNA to the transcriptional machinery [192]. In breast cancer, alterations in histone acetylation and methylation contribute to the dysregulation of gene expression and the development of aggressive malignant tumor phenotypes.

It has been reported that in the more aggressive subtypes of breast cancer, such as basal-like and TNBC, global levels of H3 lysine 27 trimethylation (H3k27me) are reduced compared to less aggressive subtypes, like luminal A and HER2-enriched, suggesting that higher H3K27me levels could be linked to a better prognosis [191,193,194]. A similar trend has been observed for H4R3me2, which is found at moderate to low levels in poor-prognosis subtypes [195]. Likewise, an increase in histone acetylation has been observed at lysine residues, such as H3K9ac (associated with HER2-enriched tumors, poor prognosis, and reduced OV), H3K18ac (associated with hormone receptor-positive malignant breast tumors), and H4K12ac (found in adjacent normal breast tissues to luminal and triple-negative malignant breast tumors) [196,197,198].

### 6.5. Immunological Biomarkers

In recent years, the emergence of new cancer treatments, such as immunotherapies that stimulate the antitumor immune response, has promoted the generation of scientific literature that analyzes the immunogenicity of breast cancer and the execution of clinical studies to identify new biomarkers that could reflect this immunogenicity and predict the response of malignant breast tumors to immunotherapy [199,200,201,202,203]. Malignant breast tumors that belong to the HER2-enriched and TNBC molecular subtypes are often highly immunogenic, while malignant breast tumors that express ER and PR hormone receptors tend to have a medium to low level of immunogenicity. Highly immunogenic malignant breast tumors that are sensitive to immunotherapy are characterized by a high expression of tumor-infiltrating lymphocytes (TILs) and programmed death-ligand 1 (PD-L1) [204,205]. In this regard, TILs and PD-L1 have emerged as potential biomarkers of immunotherapy response in breast cancer, as described below.

#### 6.5.1. PD-L1

PD-L1 is a 33 kDa type 1 transmembrane protein ligand encoded by the *PD-L1* gene (also known as *CD274* and *B7-H1*) and expressed in different types of activated immune cells, such as T lymphocytes, B lymphocytes, macrophages, and dendritic cells. PD-L1 can also be expressed on the surface of malignant tumor cells [202]. The PD-L1 ligand binds to the PD-1 receptor, encoded by the *PDCD1* gene (also known as *CD279*), which is present on T lymphocytes, B lymphocytes, natural killer (NK) cells, macrophages, and some subtypes of activated dendritic cells; as a result, PD-L1 participates in the PD-1/PD-L1 signaling pathway, which plays an important immunoregulatory role by suppressing the activation of immune cells in normal physiological contexts and in diseases, such as cancer [201,202,203,204,205]. In cancer, the interaction between the PD-L1 ligand expressed on malignant tumor cells and the PD-1 receptor located on cytotoxic T lymphocytes (CD8+) suppresses the antitumor activity of these immune cells and promotes cancer immune evasion. Therefore, PD-L1 expression contributes to tumor growth and progression primarily by inhibiting antitumor immune responses and promoting an immunosuppressive tumor microenvironment [201,202,203,204,205].

Based on the knowledge of the biological effects induced by the PD-1/PD-L1 interaction, immunotherapy drugs have been designed to block ligand–receptor binding and improve the ability of the CD8+ T lymphocytes to identify and eliminate malignant tumor cells. In this context, examples of immune checkpoint inhibitors include the anti-PD-1 monoclonal antibodies, such as Pembrolizumab and Nivolumab, and the anti-PD-L1 monoclonal antibodies, such as Atezolizumab and Durvalumab [206]. In clinical practice, the presence or absence of PD-L1 in malignant tumors is assessed by immunohistochemistry (IHC), which is the gold standard for determining which patients are eligible for immunotherapy based on immune checkpoint inhibitors [206]. For example, in TNBC, analysis of PD-L1 expression in immune and malignant breast tumor cells by IHC is a crucial step in identifying patients who may respond favorably to immunotherapy [207].

#### 6.5.2. TILs

TILs are a group of immune cells composed of lymphocytes, plasma cells, monocytes, and NK-T cells that may be scattered throughout the stroma of a malignant tumor (stromal TILs) or present within the malignant tumor (intratumoral TILs); the presence of TILs within a malignant tumor is directly related to a patient’s antitumor immune response. Based on their characteristics, TILs can be classified into cellular subgroups, such as CD8+ T lymphocytes, tissue-resident memory T lymphocytes, CD4+ helper T lymphocytes, CD4+ regulatory T lymphocytes, CD4+ follicular helper T lymphocytes, and tumor-infiltrating B lymphocytes. However, the functions of each TIL subpopulation and their clinical significance remain unknown [208,209,210].

At the pathological level, the evaluation of TILs in malignant tumor tissue should focus only on stromal TILs; this is assessed using hematoxylin and eosin staining and by analyzing the ratio between the intratumoral stromal area infiltrated by lymphocytes and plasma cells and the total intratumoral stromal area. Considering the percentage of stromal TILs present in malignant tumor tissue, three outcomes can be established: low percentage of TILs (when there are 0 to 10% stromal TILs), intermediate percentage of TILs (11 to 40%), and high percentage of TILs (greater than 40%) [210]. In this context, there is growing scientific evidence supporting the predictive and prognostic role of TILs in breast cancer. For example, in TNBC, the most immunogenic molecular subtype of breast cancer, a high percentage of TILs is associated with a better response to chemotherapy, a favorable prognosis, and prolonged survival. Finally, several studies suggest that a high percentage of TILs predicts response to neoadjuvant immunotherapy, either alone or in combination with chemotherapy, in early-stage TNBC, as well as response to immune checkpoint inhibitor-based immunotherapy in advanced or metastatic TNBC [204,208,209,210,211,212,213].

## 7. Advancements in Liquid Biopsy for the Clinical Management of Breast Cancer

In recent decades, liquid biopsy has emerged as an innovative minimally invasive technique that detects several types of molecular biomarkers, tumor cells, and metabolites in a wide variety of biological samples, such as blood and other body fluids, for the diagnosis, monitoring, and treatment of multiple cancer types, including breast cancer [214]. Conventional diagnostic techniques, such as tissue biopsy, have limited capacity to provide a comprehensive view of the dynamics of malignant breast tumor evolution; therefore, liquid biopsy offers the opportunity to conduct repeated sampling and analysis of biological samples to evaluate biomarker expression in malignant breast tumors at different disease stages, monitor tumor heterogeneity, and understand their complex biological characteristics [215,216,217,218]. Examples of liquid biopsy biomarkers are circulating tumor ctDNA, circulating tumor cells (CTCs), non-coding RNAs (ncRNAs), tumor-educated platelets (TEPs), exosomes, proteins, and metabolites; the analyses of these molecular biomarkers contribute to breast cancer’s early detection and screening, selection of the most suitable treatment modalities for patients, prognosis prediction, and monitoring of residual disease [219].

In the context of liquid biopsy for early diagnosis and clinical monitoring of breast cancer, ctDNA constitutes DNA molecules that are released by malignant breast cancer cells into the circulatory system, because of apoptosis or necrosis. It has been shown that there is a high concentration of ctDNA in plasma samples from breast cancer patients when compared to healthy counterparts; this highlights the importance of analyzing ctDNA levels for the early diagnosis of breast cancer, since its accuracy is the highest among other tumor molecular biomarkers [219,220,221]. Also, tumor-specific mutations can be identified in ctDNA samples; this shows the potential of ctDNA as a biomarker for clinical use, which can provide useful information regarding the genetic landscape of malignant breast tumors. In this case, NGS and droplet digital PCR (ddPCR) are used to evaluate the genetic alterations that drive breast cancer progression and response to treatments [215,222]. On the other hand, CTCs are breast cancer cells that detach from the primary malignant breast tumors because of the induction of the EMT process; this scenario allows them to enter the bloodstream and could reach other tissues in the body and establish metastatic colonies [223,224,225]. Since CTCs play a crucial role in metastatic dissemination, their detection in the clinical setting can provide useful insights regarding tumor progression and response to therapies [225]; clinical studies have shown that the presence of CTCs in early breast cancer patients is associated with worse OS and DFS rates [226,227,228].

## 8. Emerging Therapeutic Targets and Their Clinical Application in Breast Cancer

### 8.1. Therapies Targeting Altered Signaling Pathways in Breast Cancer

The PI3K/AKT/mTOR signaling cascade remains one of the most intensively explored therapeutic axes in breast cancer. Several inhibitors directed at this pathway have reached clinical implementation or are undergoing advanced evaluation. Among them, alpelisib is already approved for HR+/HER2- tumors harboring *PIK3CA* mutations, illustrating the relevance of molecular profiling in treatment selection [229]. Agents that block AKT, such as capivasertib, have also shown meaningful clinical benefit when paired with fulvestrant in Phase III studies [230].

Drugs targeting mTOR—specifically everolimus and temsirolimus—act predominantly on mTORC1, suppressing protein synthesis and slowing tumor cell expansion. Current research is prioritizing multi-drug regimens that incorporate endocrine therapy, chemotherapy, MAPK pathway inhibitors, or immunotherapy in an effort to mitigate resistance and improve therapeutic durability [231,232].

Resistance to pathway inhibition is frequently driven by genetic and metabolic adaptations, including PIK3CA mutations, compensatory shifts between PI3K isoforms, and broader metabolic rewiring involving glycolysis, lipid biosynthesis, or autophagy [233]. To counter these changes, several next-generation strategies are under development. These include dual PI3K/mTOR inhibitors, compounds that modulate autophagy, inhibitors of glutaminase or fatty acid synthase, and targeted degraders generated through PROTAC technology [231,234,235].

In estrogen receptor–positive tumors, Selective Estrogen Receptor Degraders (SERDs) and proteolysis-targeting chimeras (PROTACs) offer new options to disrupt or eliminate proteins that sustain tumor growth [236]. Other experimental agents, such as BIBR1591, which interfere with telomerase activity and triggers apoptosis through transcriptional alterations, exemplify how targeting specific molecular vulnerabilities may broaden therapeutic possibilities [237]. Additionally, acrolein-based delivery systems and Pro-FTY, a selective blocker of sphingosine-1-phosphate (S1P) signaling, have shown activity in multidrug-resistant models while avoiding lymphocytopenia, suggesting potential value in combination regimens [238].

As these therapeutic options evolve, progress in biomarker-guided patient selection, the incorporation of immunomodulatory strategies, and the use of AI-enhanced drug discovery and imaging technologies are expected to speed the development of more personalized oncology tools [239]. 

### 8.2. Immunotherapy and Checkpoint Inhibitors

Immunotherapy continues to introduce new avenues to strengthen antitumor responses in breast cancer. Expression of PD-L1 remains a key contributor to immune suppression within the tumor microenvironment, often limiting the efficacy of PD-1/PD-L1 blockade. However, studies combining these inhibitors with localized radiotherapy have reported improved outcomes in TNBC without notable increases in toxicity [232,240]. The identification of additional checkpoint systems, such as BTLA/HVEM, further expands opportunities to enhance immune activation and diversify therapeutic strategies [241].

This growing understanding of immune regulation reinforces the potential of combination of regimens and next-generation immunotherapies to overcome resistance mechanisms and achieve more sustained clinical responses. Parallel advances in targeted therapy complement these findings. For instance, trastuzumab deruxtecan has demonstrated superior activity compared with trastuzumab emtansine in HER2-positive metastatic disease, although careful monitoring for interstitial lung disease continues to be crucial [242]. Additional molecules under investigation include FTY720, which may reduce chemotherapy-related neuropathic symptoms while enhancing anticancer effects, and optimized clofarabine derivatives with improved potency and safety in TNBC models [243,244]. These developments collectively support a shift toward increasingly individualized therapeutic approaches.

### 8.3. Nucleic Acid–Based Therapies

Advances in RNA and DNA-based technologies have opened new perspectives for breast cancer treatment. miRNAs are of particular interest due to their ability to regulate immune checkpoint expression. Certain miRNAs—such as miR-4477a—have demonstrated both immunomodulatory and antitumor effects in breast cancer cell models [243,245]. circRNAs, exemplified by circ-ARHGER28, are also gaining attention for their diagnostic potential and their influence on signaling pathways such as PI3K/AKT/mTOR [246]. The telomerase inhibitor BIBR1591, previously mentioned, further illustrates how targeting nucleic-acid associated processes can induce apoptotic responses [237]. Moreover, CRISPR-based functional genomic screening is transforming the discovery pipeline by identifying genes involved in tumor progression and therapeutic resistance, offering a foundation for future targeted interventions—even though these tools have not yet been adapted into clinical therapies for breast cancer [247]. Furthermore, gene therapy remains a powerful frontier, aiming to correct defective genes or boost immune responses. Together, these approaches illustrate the expanding landscape of personalized and mechanism-driven therapies in breast cancer [248].

### 8.4. Nanotechnology Applied to Targeted Drug Delivery

Nanotechnology has opened new possibilities for improving targeted therapy in breast cancer. Among the most promising tools are graphene-based quantum dots, which possess favorable optical characteristics, good biocompatibility, and the capacity to enhance the precision and efficiency of drug delivery [238,249]. Recent nanosystems illustrate this potential: for example, nitrogen-doped graphene quantum dots loaded with organotin (IV) compounds have demonstrated improved solubility and a more selective delivery profile, while also offering theranostic capabilities by supporting both imaging and treatment within the same platform [238,250].

Other nanomaterials have also shown synergistic benefits. Combinations of silver graphene quantum dots with radiotherapy or agents such as 17-AAG have produced stronger anticancer responses than individual treatments, particularly through increased induction of apoptosis [251]. Additional strategies continue to emerge. One example includes hybrid nanocapsules composed of Fe_3_O_4_, chitosan, and hyaluronic acid, which have proven effective for guiding drugs or genetic material toward CD44-expressing tumor cells with high specificity [252].

Together, these advances illustrate how nanoscale delivery systems can enhance selectivity, reduce toxicity, and support the development of more precise therapeutic approaches for breast cancer.

### 8.5. Limitations in Emerging Therapeutics for Breast Cancer

Despite the progress achieved through targeted therapies, immunotherapies, and advanced molecular technologies, several challenges remain inherent to emerging therapeutic strategies. Resistance continues to be one of the most persistent obstacles, driven by the capacity of tumor cells to undergo genetic and metabolic adaptation. Furthermore, the reliance of many treatments on specific biomarkers, receptor profiles, or pathway alterations restricts their applicability to relatively narrow patient subgroups, limiting widespread clinical benefit. The heterogeneity and biological complexity characteristics of breast cancer further complicate efforts to develop interventions capable of producing consistent responses across diverse disease subtypes. Even so, advances in molecular profiling, drug design, and integrative technologies are gradually shaping a path toward more flexible and durable treatment frameworks.

Despite the growing number of innovative therapeutic approaches—ranging from pathway-targeted agents and immunotherapies to nucleic acid–based strategies and nanotechnology—current options for breast cancer still face substantial challenges. Tumor evolution, metabolic adaptability, and both inter- and intratumoral heterogeneity continue to undermine the durability of treatment responses. Moreover, many emerging therapies rely on narrowly defined biomarkers or molecular alterations, limiting their applicability to specific patient subsets and reducing the potential for broader clinical benefit. To contextualize these advances within their real-world constraints, Table 1 provides a consolidated overview of the most relevant investigational therapeutic targets, including trial phase, mechanism of action, reported adverse events, and key limitations identified to date. This synthesis highlights not only the progress made but also the critical gaps that must be addressed as these therapies move from preclinical promises toward clinical translation.

### 8.6. Clinical Application of Classic and Emerging Biomarkers in Breast Cancer

#### 8.6.1. Utility in Early Diagnosis and Risk Stratification

Across clinical guidelines, there is consensus that ER, PR, and HER2 testing are central for early breast cancer (EBC) risk assessment, with IHC and confirmatory in situ hybridization (ISH) used to classify tumors as luminal, HER2-positive, or TNBC [293,294,295,296,297,298,299]. ASCO (American Society of Clinical Oncology) highlights reporting of low ER positivity (1–10%) mandates HER2 testing and recommends multigene assays, such as Oncotype DX^®^, in ER+/HER2-, node-negative tumors to guide chemotherapy [294,295,296].

ESMO (European Society for Medical Oncology) also requires ER/PR/HER2 plus Ki-67, emphasizes preoperative endocrine response as an additional stratifier, and advises germline *BRCA1/2* testing in candidates for Poly (ADP-ribose) polymerases (PARP) inhibitors [293,298]. The Pan-Asian adapted ESMO guidelines align with these principles, emphasizing the same pathology panel, multigene signatures for HR+/HER2- EBC when chemotherapy benefit is uncertain, and *BRCA1/2* testing for both hereditary and therapeutic purposes [299]. The 2024 Spanish consensus (SEOM–SEAP) agrees on ER/PR/HER2/Ki-67 testing but extends scope with standardized HER2 scoring (0 vs. 1+) to enable T-DXd use in HER2-low disease, and formally validates several genomic assays, giving the strongest evidence to MammaPrint^®^ [297].

#### 8.6.2. Role in Therapeutic Selection and Response Monitoring

The consensus across guidelines is that biomarkers guide systemic therapy decisions in both EBC and advanced breast cancer (ABC), while the role of liquid biopsy for monitoring remains investigational (Table 2). ASCO supports testing for *PIK3CA* to identify candidates for alpelisib, *gBRCA1/2* for PARP inhibitors, and PD-L1 in TNBC for immune checkpoint inhibitor therapy; it also considers Microsatellite Instability–High (MSI-H)/Deficient Mismatch Repair (dMMR), Tumor Mutational Burden (TMB)-high, and Neurotrophic Tyrosine Receptor Kinase (NTRK) fusions actionable when matching therapies are available, but does not recommend circulating tumor DNA (ctDNA) or circulating tumor cells (CTCs) for routine monitoring [300]. ESMO advises retesting ER/PR/HER2 at metastatic diagnosis, incorporates *BRCA1/2*, *PIK3CA*, and PD-L1 as standard in ABC, and limits broader genomic profiling to situations where results would directly influence management or enable trial access, applying the ESMO Scale for Clinical Actionability of Molecular Targets, which ranks biomarkers from Tier I (standard of care) to Tier X (non-actionable) [293,298].

The Pan-Asian consensus applies these principles to regional practice, recommending ER/PR/HER2 and *gBRCA1/2* in EBC, applying multigene assays for HR+/HER2- disease when chemotherapy benefit is uncertain, and clarifying that PD-L1 should not be used in EBC; they also advise against unnecessary imaging or biomarkers unless clinically justified [299]. The Spanish consensus aligns with ASCO and ESMO on *PIK3CA*, *BRCA1/2*, and PD-L1, but distinctively includes *ESR1* mutations, often detected in ctDNA, as a biomarker to guide endocrine sequencing and the use of elacestrant after Cyclin-Dependent Kinase 4/6 inhibitor (CDK4/6i) in HR+/HER2- ABC, while similarly restricting liquid biopsy to applications in detecting therapeutic resistance rather than surveillance [297].

## 9. Current Limitations in the Clinical Assessment of Classic and Emerging Biomarkers for Breast Cancer

The clinical assessment of classic and emerging molecular biomarkers is crucial for improving the diagnosis, treatment, and monitoring of breast cancer. Current research regarding biomarkers for this disease is focused on identifying novel molecules which are characterized by their high sensitivity and specificity, plus reproducibility, to be implemented in real clinical settings. In addition, ideal biomarkers should be easily quantifiable, user-friendly, cost-effective, and measurable to ensure clinically interpretable results from readily accessible biological fluids or specimens [301]. Nevertheless, crucial limitations related to their validation, integration, standardization, and implementation in clinical practice persist in both classic biomarkers, such as Ki-67, and in emerging ones, such as PD-L1 [302].

Although Ki-67 was discovered during the 1980s and has been extensively studied since this date, its sole adoption and utility in decision-making for clinical practice remains debatable. Generally, the percentage of Ki-67 is determined by calculating the labeling index (LI) from a tissue hotspot evaluated by immunohistochemistry (IHC); however, the lack of standardization and inconsistent reproducibility related to this classic biomarker have promoted the application of complementary genomics tests, such as Oncotype Dx, MammaPrint, Endo-Predict, and Prosigna [303]. Even though the integration of Ki-67 with other diagnostic tools provides a more comprehensive biological analysis of malignant breast tumors, the implementation of additional tests increases the cost of the diagnostic process, affecting accessibility, particularly in developing countries where economic resources are limited.

Another limitation associated with the classic biomarker Ki-67 is its analytical and methodological validation. Currently, for Ki-67, there are no established criteria for the appropriate collection, fixation, and processing of breast cancer specimens during the preanalytical phase. It is worth mentioning that the clinical assessment of Ki-67 requires stricter control on the preanalytical variables, such as the type of fixative, time to fixation, and duration of fixation, as the prolonged, delayed, or insufficient fixation results in a considerable reduction in the LI [304]. Regarding analytical considerations, the main challenge is the intra- and inter-observer variability between pathologists, which is largely attributed to factors, such as intratumoral heterogeneity, difficulties in identifying a tissue hotspot region, and the lack of a well-defined measurement approach—either a score system or a counting method. Moreover, the absence of universally accepted Ki-67 cut-off values further complicates interpretation, particularly with intermediate LI. According to Mikami et al. (2013) [305], while the visual counting method shows high reproducibility, the selection of the tissue hotspot remains a critical factor, and variability becomes especially problematic in malignant breast tumors with intermediate Ki-67 levels (5–25%).

To address these limitations, the multidisciplinary group International Ki-67 in Breast Cancer Working Group (IKWG) was established in 2011, bringing together experts in pathology, medical oncology, public health, biostatistics, and biomedical research. This group aims to standardize the clinical assessment of Ki-67, through the development of evidence-based guidelines, recommendations, cut-off proposals, and training courses for pathologists. However, there is no universal regulatory framework, so the adoption of the guidelines provided by IKWG depends on each laboratory/hospital/pathologist. In parallel, the need for improved reproducibility has prompted the development of automated and semi-automated approaches for Ki-67 clinical assessment based on IKWG’s guidelines. For example, Fernezlian et al. (2023) proposed the concept of a nuclear gradient (NG) of Ki-67 categorized into NG1, NG2, and NG3/4, and analyzed through a semi-automated microscopic image approach based on staining intensity, nuclear distribution, and cell-cycle–related patterns [306]. Similarly, AI approaches have demonstrated high concordance with manual evaluation, often with improved reproducibility, reduced inter- and intra-observer variability, and greater workflow efficiency [307,308,309,310]. Importantly, the performance of AI models depends strongly on the type of algorithm employed (Convolutional Neural Networks, CNNs, or Scale-Invariant Feature Transform, SIFT), the quality of the tissue biopsy images, and the robustness of the training data [309,311]. However, both semi-automated and AI-driven strategies are still being investigated.

On the other hand, there are several limitations associated with the emerging biomarker PD-L1. For instance, currently, there is an absence of a unique standardized protocol for the analysis of PD-L1 expression in malignant breast tumor tissue [206]. Moreover, there are several molecular assays available for PD-L1 detection that include distinct antibody clones for PD-L1 evaluation; also, there are different clinically validated scoring methods for this biomarker. In this context, these tests are not directly interchangeable [206,312]. In addition, during the clinical assessment of PD-L1, interobserver variability can occur, so it is recommended that pathologists attend training sessions for PD-L1 evaluation to improve interobserver reproducibility [206]. Furthermore, it has been shown that there is a discordance in the PD-L1 expression between primary malignant breast tumors and metastatic breast tumors; this scenario highlights the importance of appropriate tissue sampling from metastatic biopsies and the urgent need for standardization of laboratory protocols for PD-L1 evaluation [313]. Nowadays, these scenarios negatively impact the ability of pathologists to correctly identify breast cancer patients who are best suited for immunotherapy based on anti-PD-L1 therapeutic approaches.

## 10. Current Challenges in the Implementation of Liquid Biopsy at the Clinical Setting for Breast Cancer Management

Despite the advantages associated with liquid biopsy, several challenges hinder its implementation in clinical scenarios to aid in the diagnosis and treatment of breast cancer patients [219]. One of the challenges is the low concentrations of molecular biomarkers, such as ctDNA, in biological samples, like blood specimens, which negatively affects the sensitivity and specificity of liquid biopsy assays; thus, it is necessary to improve the efficiency and performance of laboratory protocols for biomarker extraction and molecular detection technologies [314,315,316,317,318]. Another challenge is the lack of standardized protocols for liquid biopsy tests, which should include a consensus on aspects related to sample collection, its processing before molecular analyses, and the reporting of results; these guidelines would facilitate the implementation of liquid biopsy in the clinical setting [319,320,321,322,323,324]. In addition, a third challenge is the absence of well-designed long-term clinical studies, which would provide key insights regarding the feasibility of the application of liquid biopsy for the clinical monitoring of breast cancer cases [325].

## 11. Conclusions and Future Perspectives

Breast cancer remains a global health priority not only because of its high incidence and mortality, but also because it represents a biologically diverse set of diseases driven by layered genetic, epigenetic, and microenvironmental programs. Across this review, the evidence supports a central message: meaningful clinical progress increasingly depends on linking tumor biology to actionable biomarkers, rather than relying on clinicopathological features alone. Classic markers such as ER, PR, HER2, and Ki-67 still anchor clinical routine decision-making, enabling subtype definition, therapeutic selection, and prognostic stratification. However, their limitations—particularly variability in assessment and inconsistent cut-offs—highlight the need for better standardization, harmonized reporting frameworks, and robust quality control to ensure that biomarker information translates reliably into patient benefit.

At the same time, emerging biomarkers are reshaping how breast cancer is understood and managed. Alterations in *TP53* and EGFR, non-coding RNA networks (miRNAs, lncRNAs, circRNAs), epigenetic signatures, and immune-contexture indicators, such as PD-L1 and TILs, provide a richer description of tumor behavior, treatment sensitivity, and resistance trajectories. These candidates also reflect key biological vulnerabilities that are being exploited therapeutically through pathway inhibition (e.g., PI3K/AKT/mTOR), antibody–drug conjugates, immune checkpoint blockade, and next-generation strategies, including targeted protein degradation, nucleic-acid–based approaches, and nanotechnology-enabled delivery. Yet, the same biology that creates therapeutic opportunities also fuels failure: inter- and intratumoral heterogeneity, clonal evolution, and metabolic adaptation frequently limit the durability and generalizability of therapy responses, especially when interventions are restricted to narrowly defined molecular subgroups.

On the other hand, liquid biopsy further illustrates both promise and constraint. ctDNA, CTCs, exosomes, and circulating RNA signatures offer an appealing route to capture tumor dynamics in real time, potentially improving early detection, monitoring of minimal residual disease, and identification of therapy resistance mechanisms. Nevertheless, low analyte abundance, preanalytical variability, and the lack of universally accepted analytical and clinical validation pathways remain major barriers to routine implementation. In this setting, well-designed longitudinal studies, standardized workflows from sampling to reporting, and clinically meaningful endpoints will be essential to move liquid biopsy from an investigational tool toward a dependable component of health care.

Looking forward, the most impactful advances are likely to emerge from integrative, biomarker-guided frameworks that combine multi-omics profiling with rigorous clinical validation and equitable implementation strategies. This includes harmonizing biomarker assessment across laboratories, improving reproducibility through digital pathology and AI-assisted scoring where appropriate, and prioritizing biomarkers that are not only biologically informative but also feasible in real-world health systems. Ultimately, closing the gap between discovery and clinical utility will require coordinated efforts that connect mechanistic insight, trial design, and implementation science—so that precision oncology in breast cancer becomes both more effective and more accessible.

## Figures and Tables

**Table 1:** Summary of Novel Therapeutic Targets Being Investigated in Breast Cancer Trials.

Therapy	Drug Name	Clinical Trial Stage/Condition	Action	Adverse Effects (AE)/Limitations	Reference
**8.1 Therapies Targeting Altered Signaling Pathways in breast cancer**					
**PI3K/AKT/mTOR pathway inhibitors**	Alpelisib (BYL719) + fulvestrant	FDA-approved for HR+/HER2-treatment with PIK3CA variants (NCT02437318)	Inhibits cellular proliferation by targeting both wild-type PI3K-alpha and PI3K-alpha containing canonical variants	AE: Hyperglycemia, diarrhea, nausea, decreased appetite, rash, or maculopapular rash.	[229,231,253]
				Limitations: It is less potent against either PI3K-sigma or -gamma.	
**AKT inhibitors**	Capivasertib (AZD5363)	Phase III + fulvestrant for hormone receptor- positive and HER2 negative advanced breast cancer (ABC) with PIK3CA/AKT1/PTEN alterations (NCT04305496)	Potent inhibitor of the AKT protein (Protein Kinase B or PKB) across all three isoforms (AKT1, 2,3), blocking PI3K. AKT signaling pathway that drives cancer cell growth, survival, and metabolism	AE: Hyperglycemia, severe hyperglycemia with mixed diabetic ketoacidosis (DKA) and hyperosmolar hyperglycemic state (HHS).	[230,254,255]
				Limitation: It is CYP3A4-dependent metabolism which causes drug interactions and variability. Thus, the development of resistance through alternative signaling pathways, and the need for specific biomarkers.	
**mTOR inhibitors**	Rapamycin	Phase II for HER-2 receptor positive metastatic breast cancer-Rapamycin + Trastuzumab (NCT00411788)	Targets FKPB12/mTORC1. Inhibits lymphocyte activation and induces cell cycle arrest. Rapamycin to FKBP12 binding inhibits the activity of mTORC1 leading to a decrease in protein synthesis, increased autophagy, and inhibition of cell growth.	AE Rapamycin: Bone pain, diarrhea, headache, blurred vision, and chest pain. AE Everolimus: Loss of appetite, fatigue, diarrhea, swelling, and nausea. AE Temirolimus: Rash, itching, chest pain, hives, and flushing.	[232,256,257]
	Everolimus (RAD001) Temsirolimus (CCI-779)	Phase III for patients with breast cancer (NCT01674140) Phase III for rhabdomyosarcoma patients (NCT00703625)	Targets mTORC1. Reduces vascular endothelial growth factor (VEGF) expression and inhibits glycolysis. Inhibits mTOR activity and regulates cell division	Limitation: Suppression of mTOR triggers compensatory signaling circuits acting on upstream nodes, which in turn enhance tumor cell viability, division, and metastatic potential.	[232,240,258,259]
	AZD8055	Phase I for solid tumors and lymphoma. (Withdrawn) Phase I for breast cancer and lung cancer-AZD8055 + paclitaxel (NCT02193633)	Target both mTORC1 and mTORC2 complexes and induce cell death	AE: Increased alanine aminotransferase (22%), increased aspartate aminotransferase (22%) and fatigue (16%).	[258,260]
				Limitation: Variants in mTOR with clinical relevance increase its catalytic function and diminish the effectiveness of inhibitors.	
**Dual PI3K/mTOR inhibitors**	Gedatolisib	Phase III + fulvestrant and CDK4/6 inhibitors in individuals with HR+/HER2− locally advanced or metastatic breast cancer. (NCT03400254)	It inhibits PI3K and mTOR. Inhibits tumor growth and increase survival time.	AE: Nausea (53%), mucosal inflammation (50%), decreased appetite (40%), diarrhea (38%), fatigue (35%), and dysgeusia and vomiting (each 30%).	[231,261,262,263]
				Limitation: Resistance driven by ABC transporters (ABCB1, ABCG2), difficulty preventing metastasis from dormant cells, and side effects such as stomatitis remain concerns, even though hyperglycemia and diarrhea occur less frequently than with other PI3K inhibitors.	
**Autophagy inhibitors**	Hydroxychloroquine	FDA approved for treating malaria, rheumatoid arthritis, and lupus. Currently studying as an anticancer drug in combination with other therapies (like Palbociclib). (NCT04841148)	Increase protein acetylation. Inhibition of malignant cell growth, viability.	AE: Fatigue, nausea, diarrhea, constipation, and appetite loss.	[234,264,265]
				Limitation: Its use is constrained by poor bioavailability and broad, non-targeted distribution. In addition, HCQ’s therapeutic impact is reduced because it does not efficiently cross tumor cell membranes within the acidic tumor microenvironment	
**Glutaminase or fatty acid synthase inhibitors**	FASN inhibitor (TVB-2640)	Phase I for treatment for combinatorial treatment for breast cancer and KRAS-positive lung cancer. (NCT02223247)	It suppresses AKT phosphorylation, promotes apoptosis in tumor cells, increases the sensitivity of chemotherapy-resistant tumors to treatment, and reduces tumor growth in mouse xenograft models.	AE: Dry eyes, fatigue, dry skin, mucositis, and nauseas.	[234,266]
				Limitation: reliance on combination therapies due to limited monotherapy responses, the need to manage adverse effects such as palmar–plantar erythrodysesthesia, and strict patient eligibility criteria that exclude individuals with significant gastrointestinal or cardiac conditions.	
	Denifanstat (TVB-2640)	Phase II for relapsing high-grade astrocytoma (NCT03032484) Phase III for patients with metabolic dysfunction-associated steatohepatitis and F2/F3 fibrosis (NCT06594523)	Acts synergistically with the topoisomerase inhibitor SN-38 in TNBC brain metastasis cell lines, increases FAS expression, reduces the expression of cell-cycle–related genes, and decreases the motility of these TNBC bone marrow cells.	AE: Dry skin, dry eye and sometimes alopecia.	[234,267,268,269]
				Limitations: More studies are required for effectiveness directly on breast cancer patients. Fat-soluble aids absorption but increases off-target effects. Clinical trials have limitations such as small sample size and short treatment durations.	
**PROTACs**	Vepdegestrant (ARV-471)	Phase III for advanced metastatic breast cancer. (NCT05654623)	Block or degrade tumor-driving proteins and are associated with significantly longer progression-free survival compared with fulvestrant.	AE: Fatigue, liver enzyme elevations, nausea and hot flashes.	[263,270,271]
				Limitation: Limited oral absorption due to their size and polarity, intricate linker optimization, risks of degrading unintended proteins, delivery and cell-permeability barriers, potential overload of the proteasome, immunogenicity concerns, and difficulties in assessing pharmacokinetics and pharmacodynamics.	
**SERDs**	Fulvestrant (Faslodex)	FDA-approved for advanced ER-positive breast cancer (NCT01602380)	Binds ER in cancer cells, blocking estrogen signaling, and accelerating receptor degradation, thereby reducing tumor growth in hormone receptor–positive breast cancer.	AE: Bone pain, diarrhea, fatigue, hot flashes, nausea, and headache.	[272,273]
				Limitation: Poor water solubility, necessitating monthly intramuscular injections. Mechanism involves ER degradation, leading to potential resistance via receptor upregulation.	
	Imlunesrtant (Inluriyo)	Phase III for ER+/HER2-, and ABC (NCT04975308)	Binds to ER, particularly ERα, causing a conformational change that marks the receptor for degradation via the proteasome system, effectively eliminating it from the cell.	AE: Fatigue, decreased calcium and neutrophils, musculoskeletal pain, and increased liver enzymes (AST/ALT).	[234,274]
				Limitation: Resistance to ESR1 variants and inconsistency in brain penetration.	
**Telomerase inhibitor**	BIBR1591	In silico, In vitro, In vivo pre-clinical assessments	Induces apoptosis in altered gene expression, having anticancer effect as its expression of CDH13, DAPK1, and NR4A3 genes.	AE: Low hemoglobin/calcium/neutrophils/platelets, fatigue, musculoskeletal pain, diarrhea, nausea, constipation, elevated liver enzymes (AST/ALT), and increased cholesterol/triglycerides.	[237,275]
				Limitation: Longer treatment times (lag phase) to see effects due to telomere shortening, challenges with potency, selectivity, and drug-like properties, and potential for off-target effects as it impacts cancer cell proliferation and apoptosis	
**S1P signaling inhibitor**	Pro-FTY Fisetin	Phase II for breast cancer survivors–PROFFi (NCT06113016)	Efficient against multi-drug-resistant breast cancer. Targets S1P signaling in cancer cells, potentially affecting immune cells (lymphocytes) and tumor growth.	AE: Diarrhea, nausea, constipation, and possible liver enzyme changes.	[275,276]
				Limitation: Poor bioavailability (rapid metabolism, low solubility), requiring advanced delivery systems (nanoparticles) for better tumor targeting, and understanding its complex interaction with the Nrf2/HO-1 pathway which can promote cancer survival (careful doses consideration).	
	FTY720 (Fingolimod)	Soon to start early phase clinical trials for HER-2 positive breast cancer resistant to trastuzumab Phase III for relapsing-remitting multiple scleorosis (NCT00662649)	Induces apoptosis through ROS, suppressing survival pathways such as Akt/mTOR, and targeting cancer stem cells by lowering Oct4/Sox2/Nanog. It also limits metastasis by reducing migration and invasion (including MMP activity), disrupting cytoskeletal structures, and can act synergistically with drugs like tamoxifen or EGFR inhibitors, partly via PP2A activation and modulation of the autotaxin–LPA axis.	AE: Headache, nausea, fatigue, dizziness, cardiovascular problems, fungal infections, and enzyme elevation.	[244,277,278,279]
				Limitation: It requires in vivo phosphorylation by SphK1/2 to activate it (FTY720-P). It also has off-target effects by affecting S1P1,3,4,5 receptors and CB1. Has more side effects for its variable cellular response.	
**8.2 Immunotherapy and checkpoint inhibitors**					
**PD-1/PD-L1 inhibitors**	Pembrolizumab (Keytruda) Targeting PD-1	Phase III for triple-negative breast cancer. (NCT02819518) Expression of PD-L1 in ER/PR negative breast tumors (NCT03197389)	Blocks the PD-1 receptor on T cells, preventing cancer cells from using the PD-L1/PD-L2 pathway to evade the immune system, thereby allowing the body to attack the tumors.	AE: Diarrhea, nausea, fatigue, pain, rash, itching, cough, and fever.	[280,281]
				Limitation: It is ineffective in tumors with low PD-L1 expression and can develop resistance even in those with high PD-L1 levels. It may also overstimulate the immune system as an off-target effect.	
	Atezolizumab (Tecentriq) Targeting PD-L1	Phase IIIb for advanced or metastatic PD-L1-positive triple-negative breast cancer (NCT04148911)	Blocks the PD-L1 protein on cancer and immune cells, preventing it from binding to PD-1 on T cells. This keeps the immune response from being switched off, allowing the immune system to attack the tumor.	AE: Fatigue, cough, decreased appetite, difficulty in breathing, anemia, constipation, fever, and diarrhea.	[282,283]
				Limitation: It is susceptible to resistance through alternative immune checkpoints (such as CTLA-4), tumor heterogeneity in PD-L1 expression, poor T-cell infiltration, or the development of an immunosuppressive tumor microenvironment (e.g., expansion of myeloid-derived suppressor cells).	
**Immunotherapy**	Trastuzumab deruxtecan (T-DXd)	Phase III for HER2-positive metastatic breast cancer, and first-line metastatic breast cancer (NCT04784715)	It is an antibody–drug conjugate (ADC) that links trastuzumab (which targets the HER2 protein) to a potent chemotherapy payload (deruxtecan). This design allows it to kill HER2-positive cancer cells and, through a “bystander effect,” also nearby tumor cells, improving treatment outcomes in HER2-positive and HER2-low breast cancer and other solid tumors.	AE: Fatigue, constipation, diarrhea, alopecia, neutropenia, increased liver enzymes, and anemia.	[242,284,285]
				Limitation: It has challenges in predicting the response it would generate. It is not immune to resistance mechanisms of cancer cells like decreased HER2 expression, altered ADC internalization, and issues with payload release/action (like CTSL activity).	
**8.3 Nucleic Acid-Based Therapies**					
**miRNA therapy**	miR-4477a	Pre-clinical assessments in co-culture in vitro breast cancer cell line.	They bind to three separate regions of PD-L1 mRNA with high affinity (94%, 88%, and 80%), allowing them to effectively target and suppress key regulatory pathways essential for cancer cell function.	AE: No clinical trial performed yet, so there are any reported.	[243,245,286]
				Limitation: It has challenges in delivery efficiency, understanding complex roles in cell cycles, and instability. Likewise, there are no clinical trials performed, so the is limited information.	
**Circular RNAs therapy**	circ-ARHGER28	Pre-clinical assessments on MCF-7 cells for breast cancer.	It functions as a tumor suppressor in breast cancer by limiting cell proliferation and inducing apoptosis, mainly through inhibition of the PI3K/AKT/mTOR pathway, ultimately slowing tumor growth and highlighting its therapeutic potential. It promotes cisplatin resistance in ovarian cancer.	AE: No clinical trial performed yet, so there are any reported.	[246,287]
				Limitation: It has been evidence of resistance to treatment in pre-clinical trials by genome evolution of cancer cells. There is still limited access to information.	
**CRISPR-based technologies**	Disrupting ITGA9 (Integrin Alpha 9)	Pre-clinical assessments on mouse models.	Inhibits or reduces the ITGA9 function, often using antagonists, shRNA, or siRNA, to block its role in cell adhesion, migration, and inflammation.	AE: No clinical trial performed yet, so there are any reported.	[247,248,288,289]
				Limitation: It involves complex pathway compensation (like other integrins, α5β1), context-dependent effects (strong in inflammation/angiogenesis), and varied mechanisms for achieving depletion (gene loss vs. epigenetic silencing). There are complex interactions that need understanding and limited studies.	
	Silencing SRC-1	Pre-clinical assessments in ex vivo metastatic tumors of endocrine-treated human breast cancer.	Suppressing SRC-1 reduces metastatic spread in breast cancer, even though it does not significantly affect primary tumor growth.	AE: No clinical trial performed yet, so there are any reported.	[247,290,291]
				Limitation: SRC-1 is suppress but it is a key coactivator for hormone receptors, nuclear organization, and multiple signaling pathways. Thus, disrupting SRC-1 can interfere with these essential functions, causing altered cellular responses.	
**8.4 Nanotechnology Applied to Targeted Drug Delivery**					
**Graphene quantum dots**	Graphene quantum dots + Pembrolizumab	Pre-clinical assessments in in vivo human breast cancer imaging.	Provide effective and targeted delivery with enhanced solubility, reduced toxicity, and theragnostic potential by enabling simultaneous treatment and imaging. High tumor activity and specific targeting using a radiolabeled probe.	AE: No clinical trial performed yet, so any reported.	[249,292]
				Limitation: It has complex synthesis, and unknown long-term biodistribution. There is limited access to information and studies.	

**Table 2:** Comparison of biomarker recommendations in breast cancer across major clinical guidelines.

Routine Clinical Use (Standard of Care)			
**ESMO** [293,298]	**ASCO** [300]	**Pan-Asian** [299]	**Spanish** [297]
ER, PR, HER2 (IHC ± ISH) for all invasive cancers; re-test in MBC	ER, PR (IHC) for all invasive cancers	ER, PR (IHC) for all invasive cancers	ER, PR, HER2, Ki-67 (IHC ± ISH) in all EBC
Ki-67 as part of initial risk assessment	HER2 (IHC/ISH) for all invasive cancers	HER2 (IHC ± ISH) for all invasive cancers	HER2-low reporting (0 vs. 1+) to enable T-DXd
Multigene assays (HR+/HER2- EBC, uncertain chemo benefit)	Oncotype DX (HR+/HER2-, node-negative)	Validated multigene assays (HR+/HER2- EBC)	Oncotype DX^®^, MammaPrint^®^, Prosigna^®^, EndoPredict^®^ in ER+/HER2- EBC
PD-L1 testing in metastatic TNBC	PD-L1 testing in TNBC	–	PD-L1 testing in metastatic TNBC
*PIK3CA* testing in HR+/HER2- MBC	*PIK3CA* mutations → alpelisib	–	*PIK3CA* testing in HR+/HER2- ABC
gBRCA1/2 in EBC (adjuvant olaparib) and in HER2- MBC (PARP inhibitors)	g*BRCA1/2* mutations → PARP inhibitors	g*BRCA1/2* testing for adjuvant olaparib	g*BRCA1/2* in high-risk HER2- EBC and advanced disease
MSI-H/dMMR, TMB-high for immunotherapy	Same	–	–
NTRK fusions for TRK inhibitors	Same	–	–
–	–	–	*ESR1* mutations (ctDNA) to guide endocrine therapy (HR+/HER2- ABC).
**Research/Limited or optional use**			
**ESMO** [293,298]	**ASCO** [300]	**Pan-Asian** [299]	**Spanish** [297]
*ESR1*, somatic *BRCA*, HER2-low (optional)	*ESR1* (emerging)	–	–
MSI-H/dMMR, TMB-high, NTRK (only if matched drugs available)	Same	Same	Same
TILs (prognostic; no treatment cut-offs)	Same	Same	Same
TROP2 (investigational for ADCs)	Same	–	Not required for sacituzumab govitecan
*PALB2* (possible PARP use, not established)	Same	–	–
ctDNA/CTCs (prognostic, not for therapy decisions)	Same	–	–
–	–	HRD beyond BRCA, AKT-pathway, broad NGS (investigational)	Same
**Not recommended**			
**ESMO** [293,298]	**ASCO** [300]	**Pan-Asian** [299]	**Spanish** [297]
PD-L1 in EBC	Routine HRD testing outside BRCA	PD-L1 in EBC (not predictive in neoadjuvant TNBC)	Routine MSI-H/dMMR testing (rare in breast cancer)
Broad genomic profiling or ctDNA when not actionable	Routine TROP2 testing	Routine lab tumor markers/extensive imaging for all	Routine broad NGS for off-label therapy
–	Routine ctDNA/CTCs for monitoring	–	PD-L1 for EBC (benefit in TNBC regardless of PD-L1)

## Data Availability

No new data were created or analyzed in this study. Data sharing is not applicable to this article.

## Abbreviations

The following abbreviations are used in this manuscript:

ABC	Advanced breast cancer
ADC	Antibody–drug conjugate
AE	Adverse effects
AR	Androgen receptor
AREG	Amphiregulin
ASCO	American Society of Clinical Oncology
CD8+	Cytotoxic T lymphocytes
CDK4/6i	Cyclin-Dependent Kinase 4/6 inhibitor
cDNA	Complementary DNA
circRNAs	Circular RNAs
CTCs	Circulating tumor cells
ctDNA	Circulating tumor DNA
D	Hinge region
DBD	DNA-binding domain
DCIS	Ductal carcinoma in situ
DFS	Disease-free survival
ddPCR	Droplet digital PCR
dMMR	Deficient Mismatch Repair
EBC	Early breast cancer
ECD	Extracellular domain
EGFR/HER1/ERBB1	Epidermal growth factor receptor
EMT	Epithelial–mesenchymal transition
ER	Estrogen receptors
ESCAT	ESMO Scale for Clinical Actionability of molecular Targets
ESMO	European Society for Medical Oncology
ESR1	Estrogen Receptor 1
FM-miR-34a	Fully modified version of miR-34a
gBRCA1/2	Germline BRCA1/2
H3k27me	H3 lysine 27 trimethylation
HATs	Histone acetyltransferases
HBEGF	Heparin-Binding EGF-Like Growth Factor
HDACs	Histone deacetylases
HDI	Human Development Index
HDR	Homologous Recombination Deficiency
HER2	Growth factor receptor-2
HER2/ERBB2	Human epidermal growth factor receptor-2
HMTs	Histone methyltransferases
HOTAIR	HOX transcript antisense intergenic RNA
HR+	Hormone receptor-positive
IHC	Immunohistochemistry
IKWG	International Ki67 in Breast Cancer Working Group
ISH	In Situ Hybridization
LAR	Luminal androgen receptor
LDB	Ligand-binding domain
LI	Labeling index
lncRNAs	Long non-coding RNAs
MBC	Metastatic Breast Cancer
miRNAs	MicroRNAs
MSI-H	Microsatellite Instability–High
ncRNAs	non-coding RNAs
NGS	Next-generation sequencing
NK	Natural killer cells
NTRK	Neurotrophic Tyrosine Receptor Kinase
oncomiRs	Oncogenic miRNAs
OS	Overall survival
PARP	Poly (ADP-ribose) polymerases
PD-L1	Programmed death-ligand 1
PIK3CA	Phosphatidylinositol-4,5-Bisphosphate 3-Kinase Catalytic Subunit Alpha
PR	Progesterone receptors
PROTACs	Proteolysis-targeting chimeras
PRS	Polygenic risk score
RTKs	Receptor tyrosine kinases
S1P	Sphingosine-1-phosphate
SERDs	Selective Estrogen Receptor Degraders
SNPs	Single nucleotide polymorphisms
TAD	Transactivation domain
TCGA	Cancer Genome Atlas
T-DXd	Trastuzumab deruxtecan
TEPs	Tumor-educated platelets
TILs	Tumor-infiltrating lymphocytes
TMB	Tumor Mutational Burden
TMD	Transmembrane domain
TNBC	Triple-negative breast cancer
TROP2	Trophoblast Cell-Surface Antigen 2
VEGF	Vascular endothelial growth factor
WHO	World Health Organization
